# Supplementary material for: Sustainable thermal regulation improves stability and efficiency in all-perovskite tandem solar cells
Source: Nat Commun. 2024 May 16;15:4136. doi: 10.1038/s41467-024-48552-2 (PMC11099067; doi:10.1038/s41467-024-48552-2)
Supplement: Supplementary file 1 — Supplementary Information [file 41467_2024_48552_MOESM1_ESM.pdf]

Supplementary Information

Sustainable thermal regulation improves stability and efficiency in all-perovskite tandem solar cells

Contents

Supplementary Note 1 4
Supplementary table I 5
Supplementary Figure 1 6
Supplementary Figure 2 7
Supplementary Figure 3 8
Supplementary Figure 4 9
Supplementary Figure 5 10
Supplementary Figure 6 11
Supplementary Figure 7 12
Supplementary Figure 8 13
Supplementary Figure 9 14
Supplementary Figure 10 15
Supplementary Figure 11 16
Supplementary Figure 12 17
Supplementary Figure 13 18
Supplementary Figure 14 19
Supplementary Figure 15 20
Supplementary Figure 16 21
Supplementary Figure 17 22
Supplementary Figure 18 23
Supplementary Figure 19 24
Supplementary Figure 20 25
Supplementary Figure 21 26
Supplementary Figure 22 27
Supplementary Figure 23 28
Supplementary Figure 24 29
Supplementary Figure 25 30
Supplementary Figure 26 31

Supplementary Figure 27 32
Supplementary Figure 28 33
Supplementary Tables 34
References 40

Supplementary Note 1

The DFT Computational details.

All DFT calculations were implemented using the Vienna ab initio Simulation Package (VASP).[1-3] The electronic exchange and correlation were represented by the generalized gradient-corrected Perdew−Burke−Ernzerhof functional method (GGA-PBE).[4, 5] A plane-wave cutoff energy of 400 eV was used for the electronic wave function in all computations. The Brillouin zone was sampled by (2×2×1) and (4×4×1) Monkhorst-Pack k-point mesh for geometries relaxed and electronic structure calculations respectively.[6] To avoid the interaction between adjacent layers, a vacuum layer of 20 Å was established in the z-axis direction. The convergence criteria for energy and force were set to be 10^{-4} eV and 0.05 eV/Å. The dipole moment correction was turned on. The van der Waals forces in the structure were considered and the correction was achieved using Grimme's DFT-D3 method.[7] The charge calculation was implemented using the Bader charge method developed by Henkelman and coworkers.[8]

DFT calculation of isobaric molar specific heat capacity of *ortho*-Carborane.

The molecular structure of *o*-CB was optimized under B3LYP/6-311g(d) level with the dispersion correction of GD3BJ by the software Gaussian 16 Reversion A 03 and confirmed without any imaginary frequencies. All the data have been listed in Supplementary Table I. The isobaric molar specific heat capacity of *o*-CB was calculated in different temperatures under 1 air pressure by the tool named freqchk in Gaussian 16 Reversion A 03 software without any correction factors.

Supplementary table I

Symbol	NA	NB	NC	Bond	Angle	Dihedral	X	Y	Z
B							-1.388506	-0.590919	0.760986
B	1			2.8839124			0.796279	1.291473	0.744697
B	1	2		1.7827418	71.9983236		0.135373	-1.515744	0.735302
B	1	3	2	1.7631701	59.6460759	100.9905816	-0.79674	-1.291556	-0.744899
B	3	1	4	1.7628565	108.2033263	37.9230518	0.980262	-1.14343	-0.766433
B	5	3	1	1.7628729	60.7313118	100.6146829	1.485438	-0.352181	0.725694
B	1	4	3	1.7822611	108.2078318	100.6315143	-0.98011	1.143912	0.766356
B	1	4	3	1.7627656	60.7257826	138.0310149	-1.485844	0.35199	-0.725214
B	7	1	8	1.7630212	108.1971431	-37.9130095	-0.135133	1.515268	-0.73576
C	9	7	1	1.7032873	103.9213709	-2.2690479	-0.019738	0.009768	-1.524012
H	2	1	8	1.1802498	143.5290413	126.9522889	1.321798	2.141049	1.373219
H	3	1	8	1.179803	119.8201335	-154.8562063	0.226352	-2.514048	1.357433
H	5	3	1	1.1800382	126.1836979	-143.9262157	1.622837	-1.895974	-1.40929
H	6	5	3	1.1801294	126.2084575	106.559614	2.465001	-0.585166	1.341226
H	1	8	10	2.3978499	123.5660352	73.1452565	0.03335	-0.016931	2.604501
H	7	1	8	1.1801767	119.8262753	116.9625081	-1.623242	1.896357	1.409026
H	9	7	1	1.18049	126.1933729	144.0109188	-0.22565	2.514465	-1.357828
H	10	9	7	2.5188473	72.4621916	-126.7271233	2.300789	0.981591	-1.400293
H	10	9	7	1.0805048	117.1438812	148.578225	-0.034226	0.01661	-2.604398
H	1	8	10	1.1801443	126.1981435	-155.1057648	-2.30083	-0.980881	1.399997
H	8	1	4	1.1801258	126.2823181	106.5664421	-2.464935	0.585352	-1.341347
H	4	1	8	1.1801027	126.2562738	-106.6297361	-1.321778	-2.141642	-1.372857
C	1	8	10	1.7038453	103.9356452	58.6342299	0.01965	-0.009766	1.524168
B	10	9	7	1.7045401	63.0878231	-103.0471296	1.389194	0.591029	-0.760793
	10	9	7	1.5241741	62.8583068	-31.3919140	0	0	0

Supplementary Figure 1

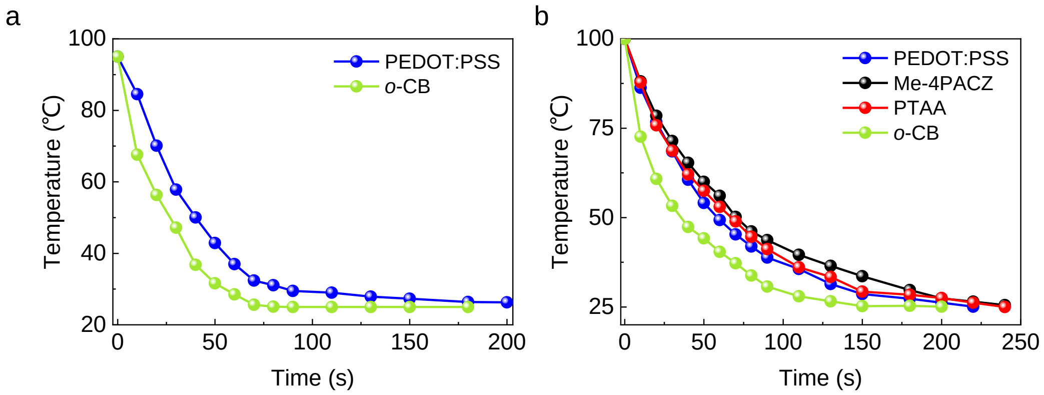

Supplementary Figure 1. a, Variation of surface temperature with time for *o*-CB (1 mg mL^{-1}) and PEDOT:PSS (13 mg mL^{-1}) in concentration of practical applications from 100 °C to 25 °C. Cooling rate: PEDOT:PSS$_{rate}$ = 0.318 °C/s; *o*-CB$_{rate}$ = 0.875 °C/s. b, Variation of surface temperature with time for *o*-CB (13 mg mL^{-1}), Me-4PACZ (13 mg mL^{-1}), PTAA (13 mg mL^{-1}), and PEDOT:PSS (13 mg mL^{-1}) from 100 °C to 25 °C. Cooling rate: PEDOT:PSS$_{rate}$ = 0.341 °C/s; PTAA$_{rate}$ = 0.3125 °C/s ; Me-4PACZ$_{rate}$ = 0.31 °C/s; *o*-CB$_{rate}$ = 0.5 °C/s.

Supplementary Figure 2

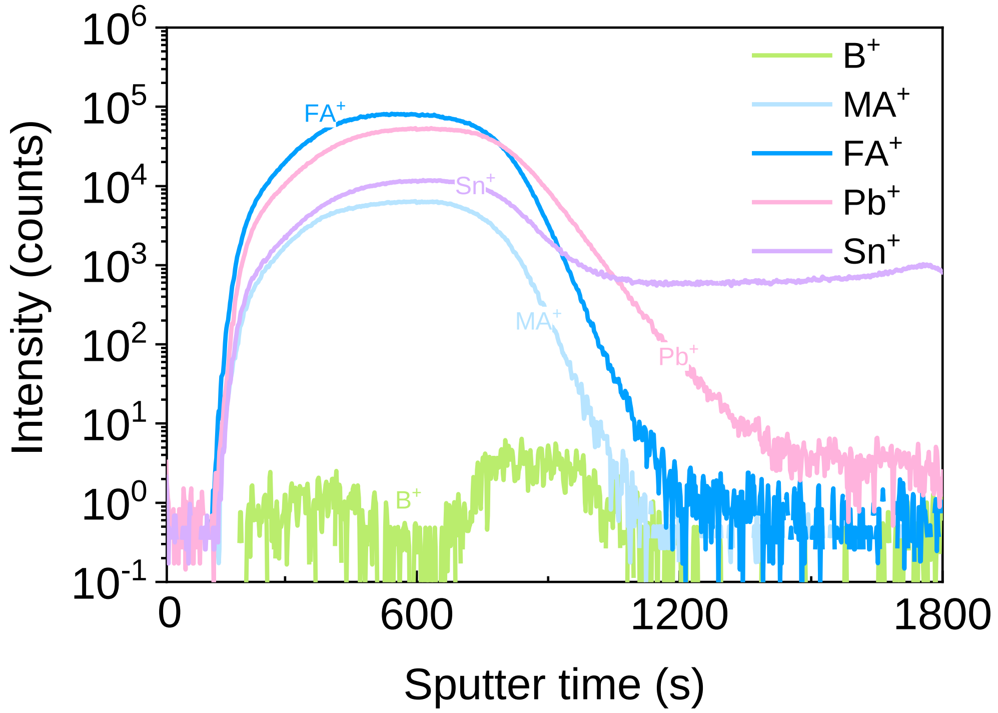

Supplementary Figure 2. Confirmation of the distribution of elements by Time-of-flight secondary ion mass spectrometry (TOF-SIMS).

Supplementary Figure 3

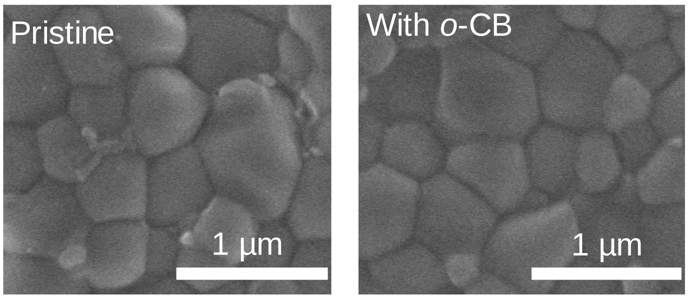

Supplementary Figure 3. SEM of pristine film and treated with *o*-CB before heating at 85 °C.

Supplementary Figure 4

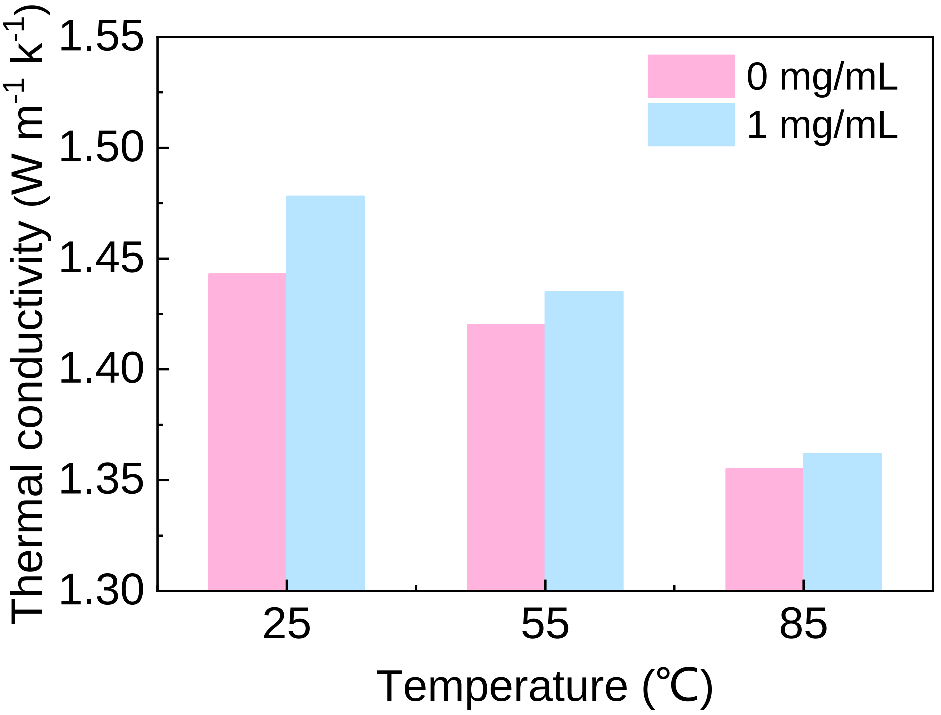

Supplementary Figure 4. Thermal conductivities of the complete devices with perovskite films treated with/without *o*-CB.

The thermal conductivities of the pristine devices were 1.443 W m^{-1} k^{-1}, 1.42 W m^{-1} k^{-1}, and 1.355 W m^{-1} k^{-1} at 25 °C, 55 °C, and 85 °C. In contrast, the thermal conductivities of devices with *o*-CB treatment increased to 1.478 W m^{-1} k^{-1}, 1.435 W m^{-1} k^{-1}, and 1.362 W m^{-1} k^{-1} at 25 °C, 55 °C, and 85 °C.

Supplementary Figure 5

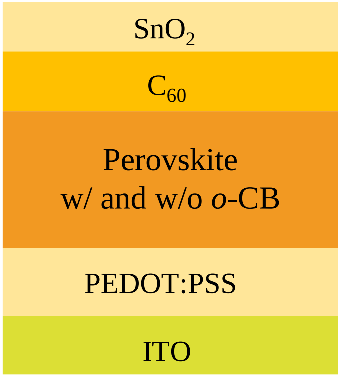

Supplementary Figure 5. The photothermal response of each layer in PSCs with the architecture.

Supplementary Figure 6

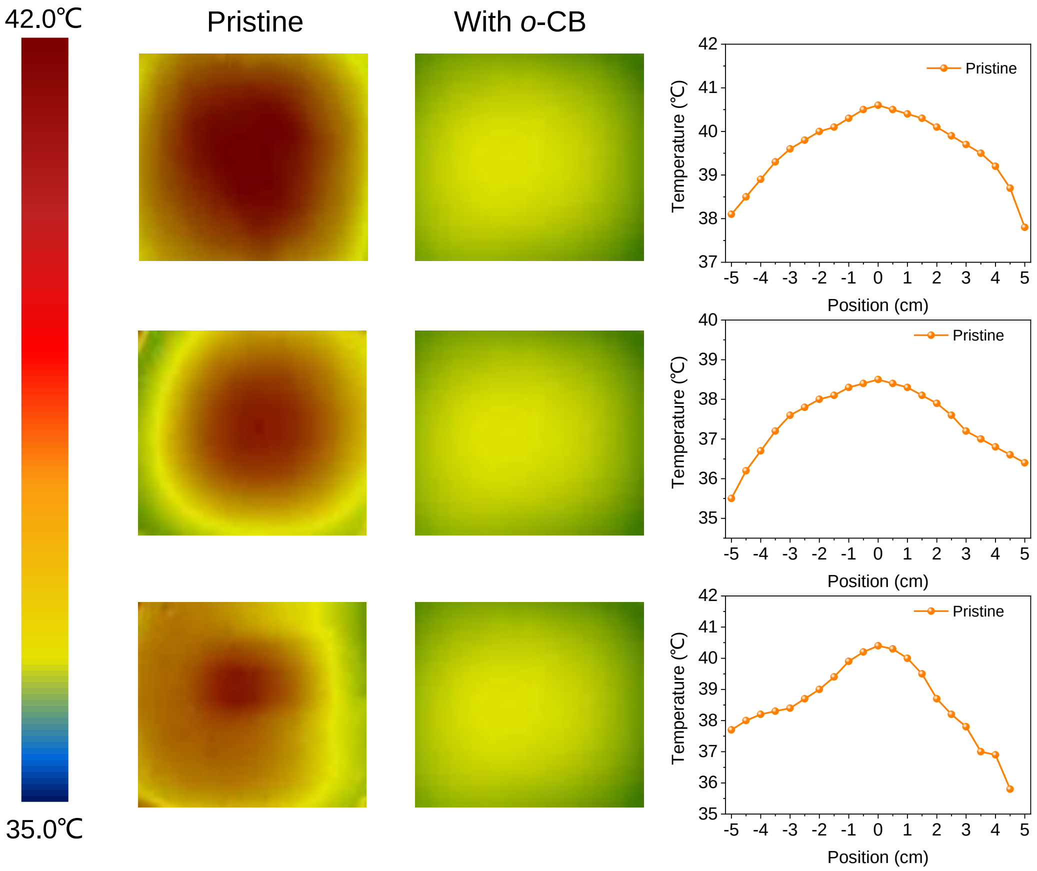

Supplementary Figure 6. IR thermal images of pristine perovskite film (5 cm * 5 cm) and perovskite film with *o*-CB treatment (5 cm * 5 cm) under 1-sun illumination for 2000 s.

The pristine film after 2000s irradiation showed strong temperature concentration, and the statistics showed that the temperature was mostly concentrated in the middle part of ITO and unevenly distributed.

Supplementary Figure 7

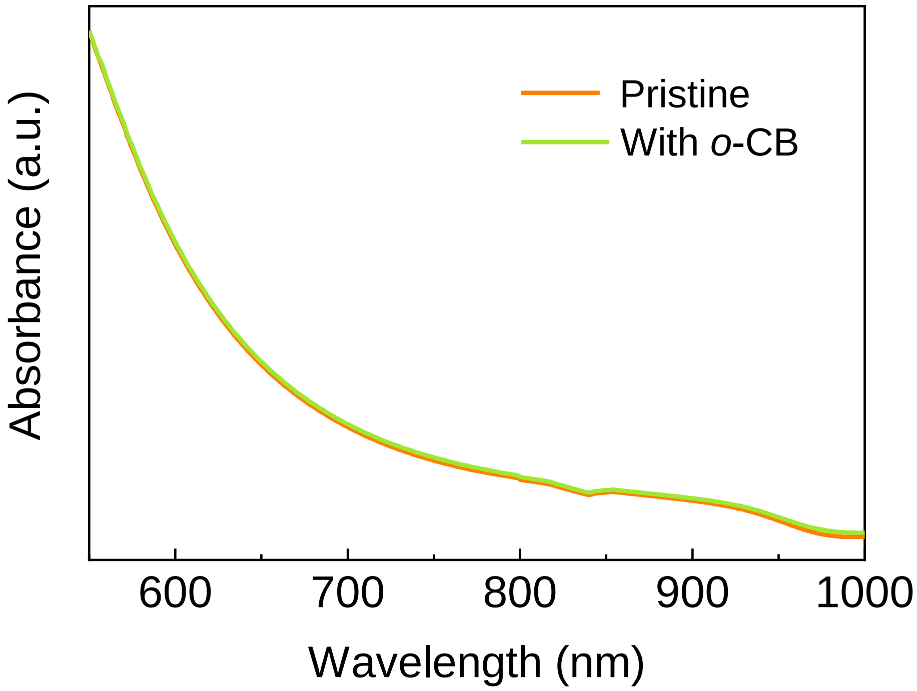

Supplementary Figure 7. UV-Vis spectra of perovskite films.

The absorbance of the perovskite films did not change significantly after the introduction of *o*-CB.

Supplementary Figure 8

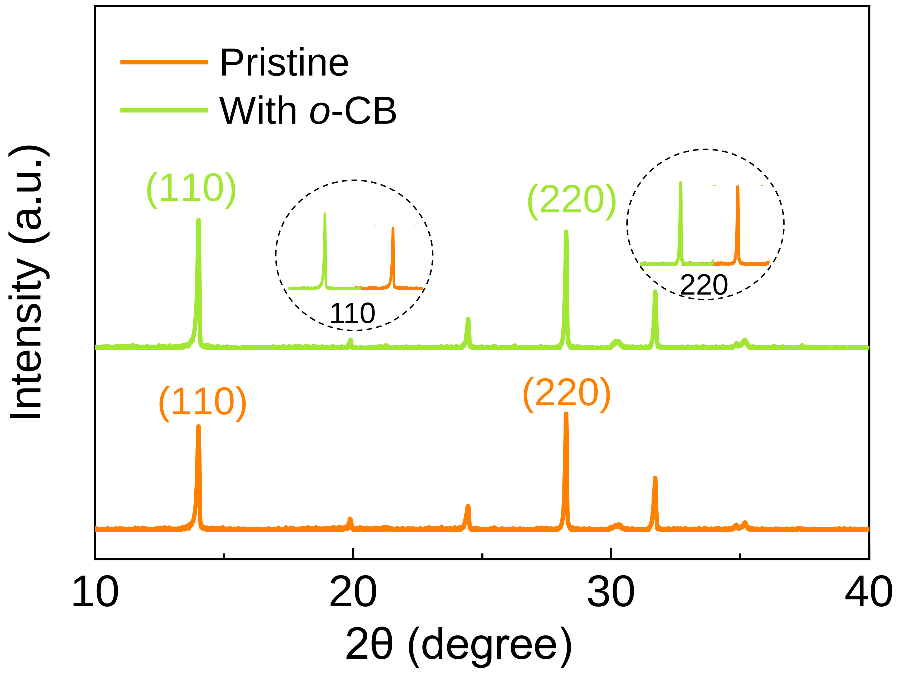

Supplementary Figure 8. XRD spectra of the perovskite film w/ and w/o *o*-CB treatment.

.

Supplementary Figure 9

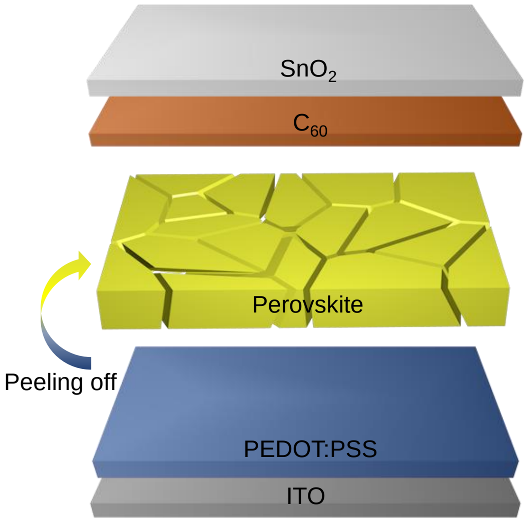

Supplementary Figure 9. Schematic structure of perovskite film stripped from PEDOT: PSS with solidified glue.

Supplementary Figure 10

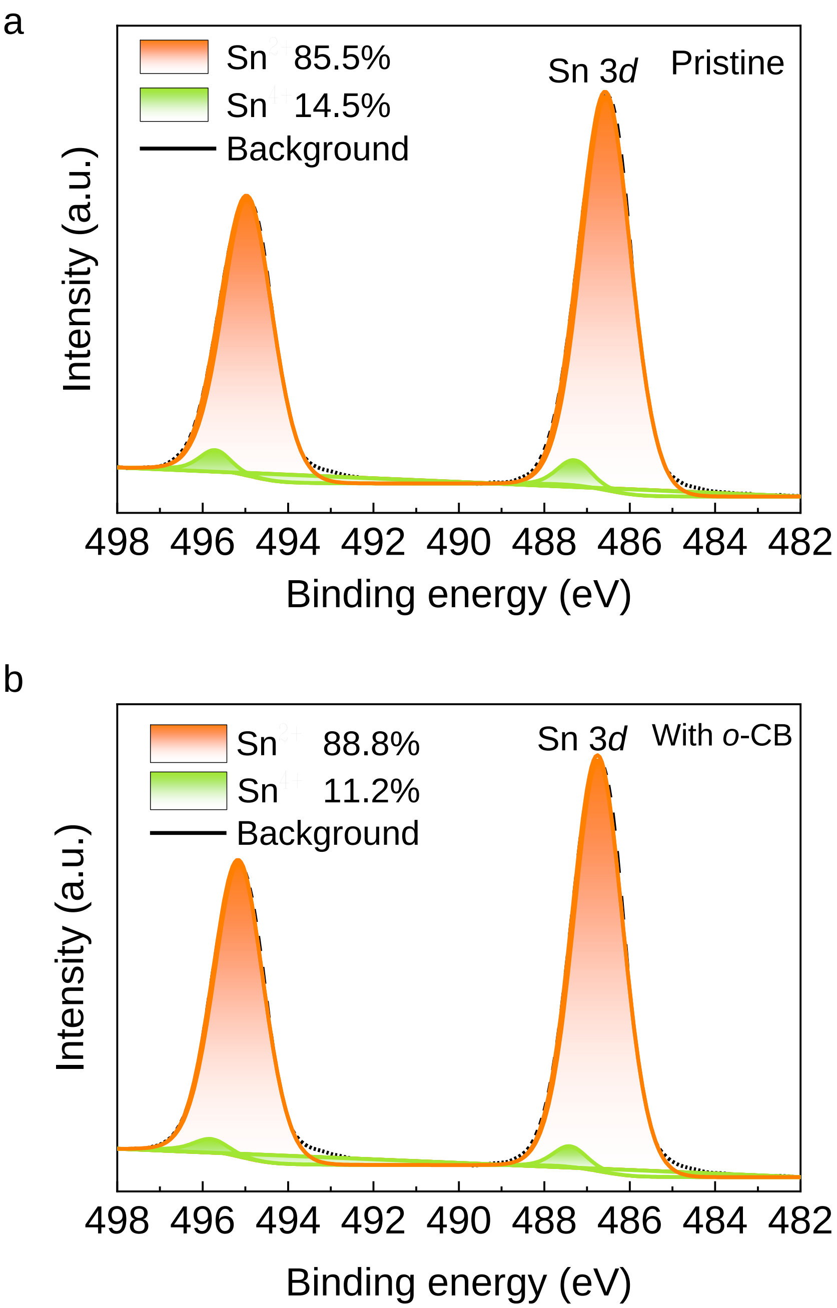

Supplementary Figure 10. XPS spectra for Sn 3*d* orbit of pristine perovskite film **a)** and perovskite film treated by *o*-CB **b**).

The reduction of Sn^{4+} content in the *o*-CB treated film compared to the pristine film is more visualized by the peak area ratio. The reduction of Sn^{4+} content may be related to the template effect of the *o*-CB regulating the growth of perovskite film, which reduces the intrusion of oxygen and makes the film more stable.

Supplementary Figure 11

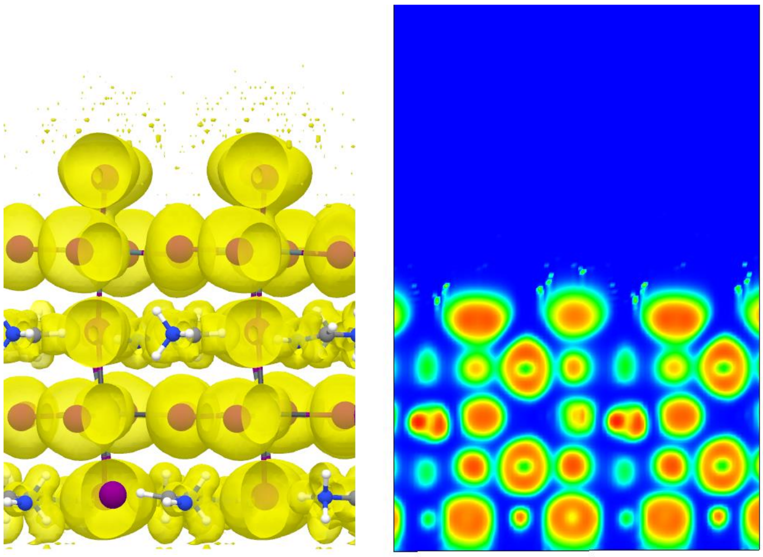

Supplementary Figure 11. The electronic location function diagram of pristine perovskite.

Supplementary Figure 12

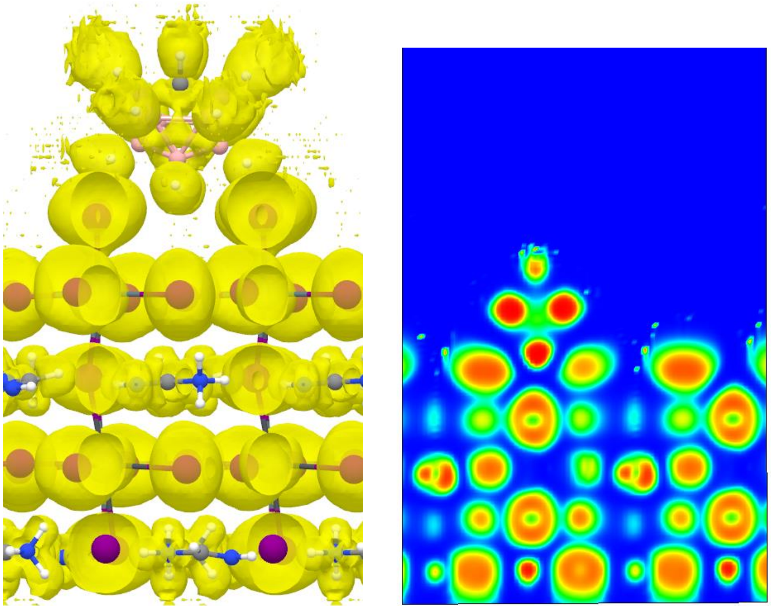

Supplementary Figure 12. The electronic location function diagram of perovskite treated with *o*-CB.

Supplementary Figure 13

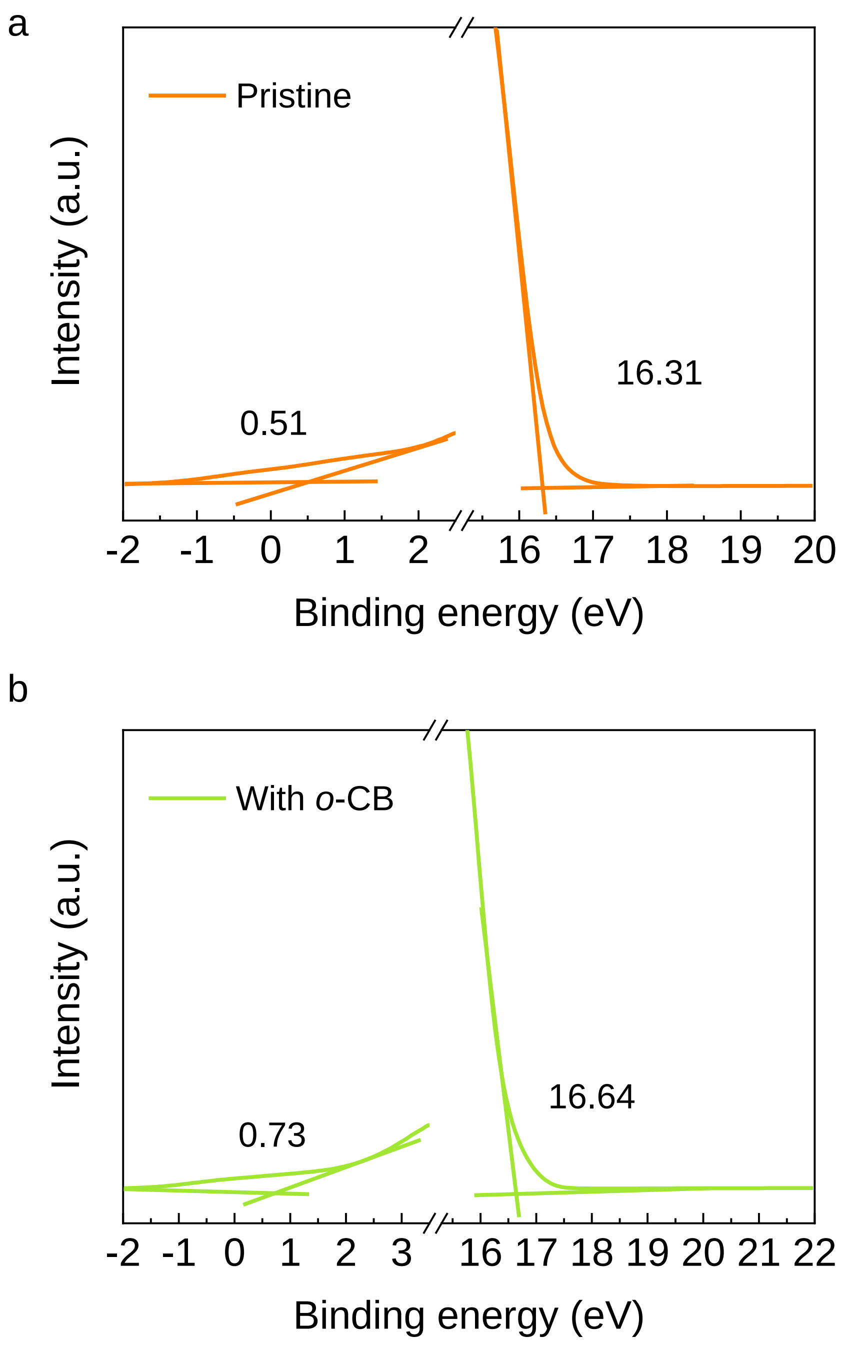

Supplementary Figure 13. UPS spectra of pristine perovskite film **a)** and perovskite film treated by *o*-CB **b**).

Supplementary Figure 14

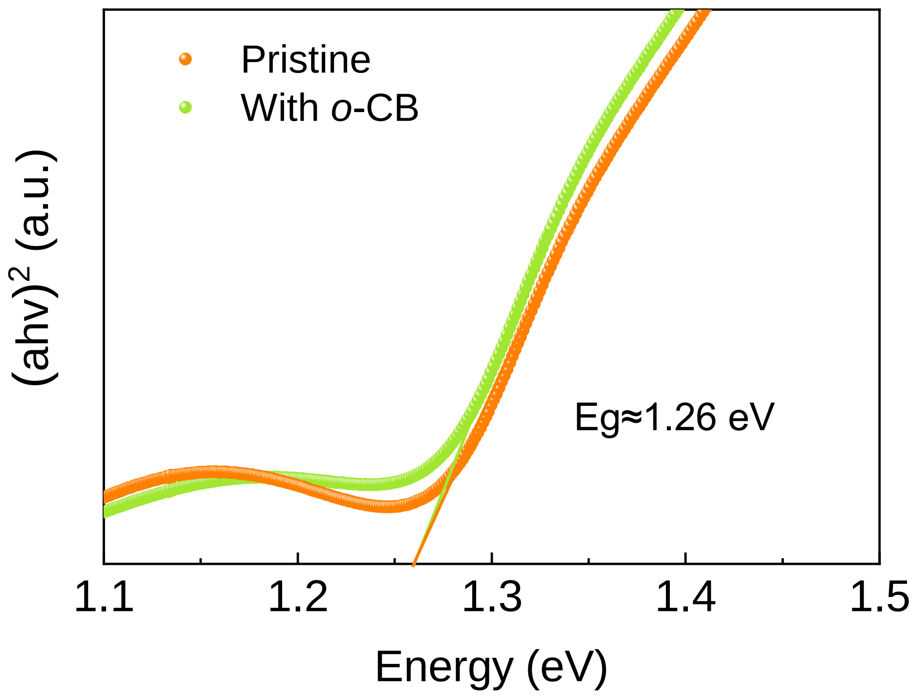

Supplementary Figure 14. Bandgap calculation from Tauc plots of the perovskite films.

The band gap value was confirmed by the Tauc plot tangent to the x-axis intercept, and the band gap value of the perovskite film remains unchanged after the treatment by *o*-CB.

Supplementary Figure 15

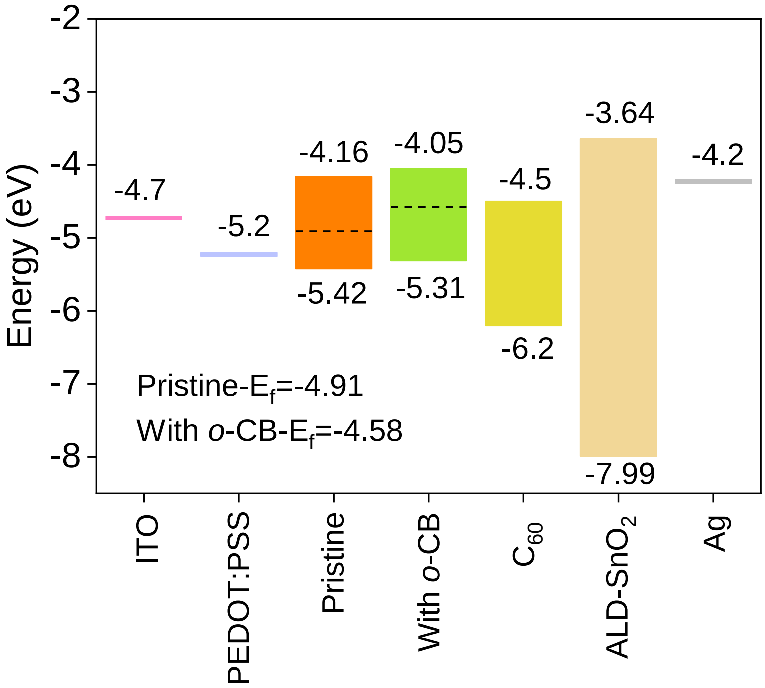

Supplementary Figure 15. Energy level arrangement of the complete device.

Supplementary Figure 16

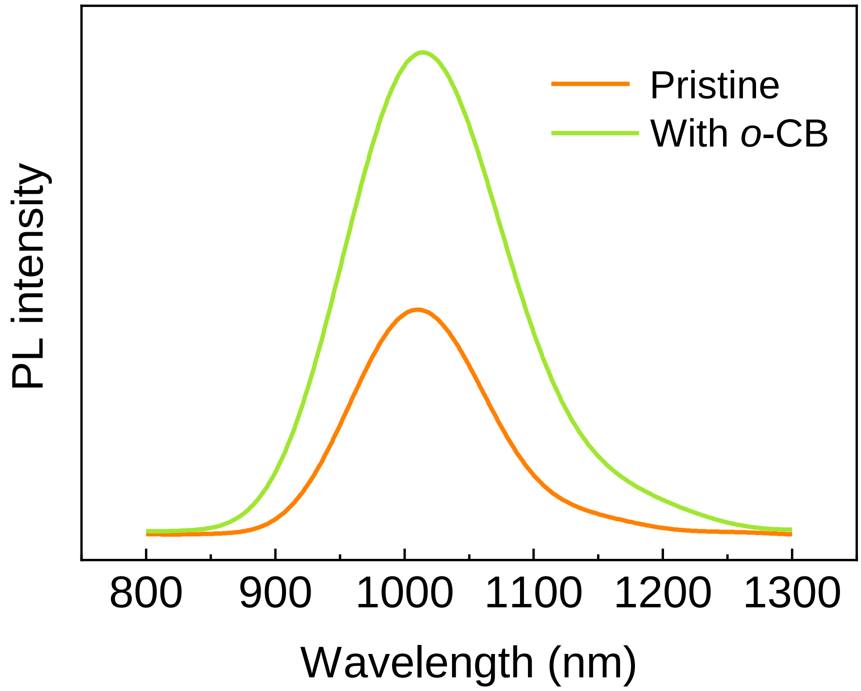

Supplementary Figure 16. PL spectra of perovskite films.

The PL intensity of the film after *o*-CB treatment was significantly stronger than that of the pristine film, proving that the non-radiative recombination of the film treated by *o*-CB was effectively inhibited

Supplementary Figure 17

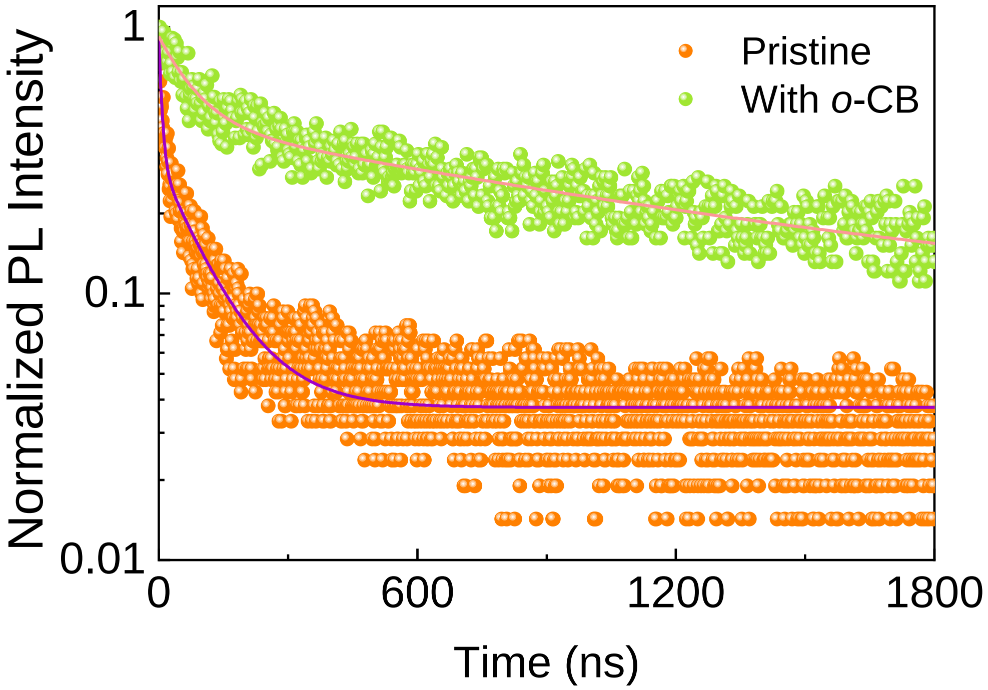

Supplementary Figure 17. The time-resolved PL spectra of the perovskite films.

The average carrier lifetime of the perovskite film treated by *o*-CB increased significantly (from 358 ns for the pristine film to 630 ns for the perovskite film treated by *o*-CB), indicating that the improved interfacial trap states and the inhibition of non-radiative recombination.

Supplementary Figure 18

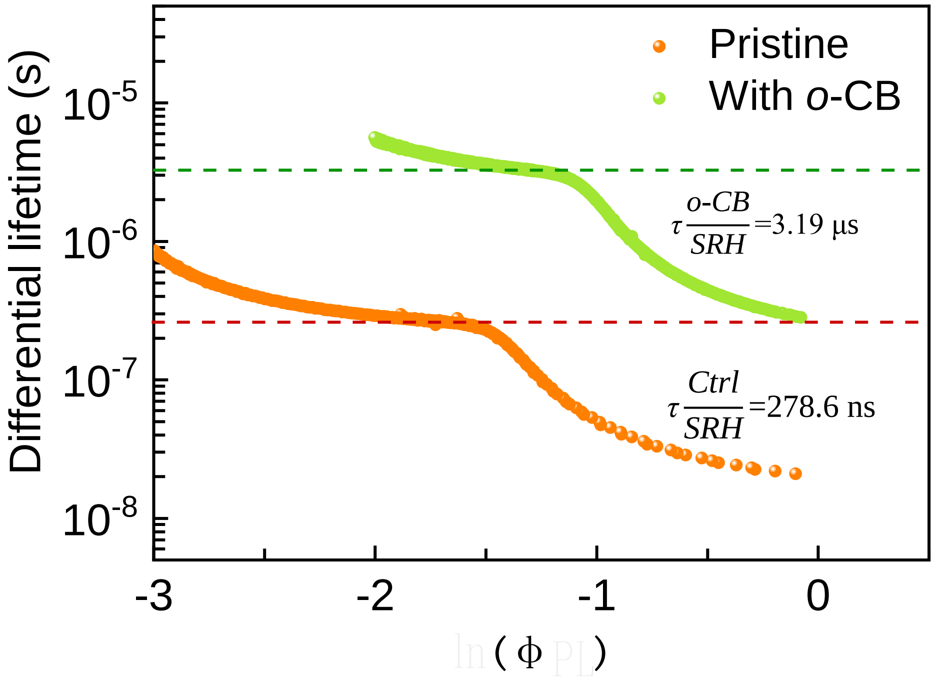

Supplementary Figure 18. Differential lifetime versus the logarithm of the PL intensity (ln(ΦPL)).

Supplementary Figure 19

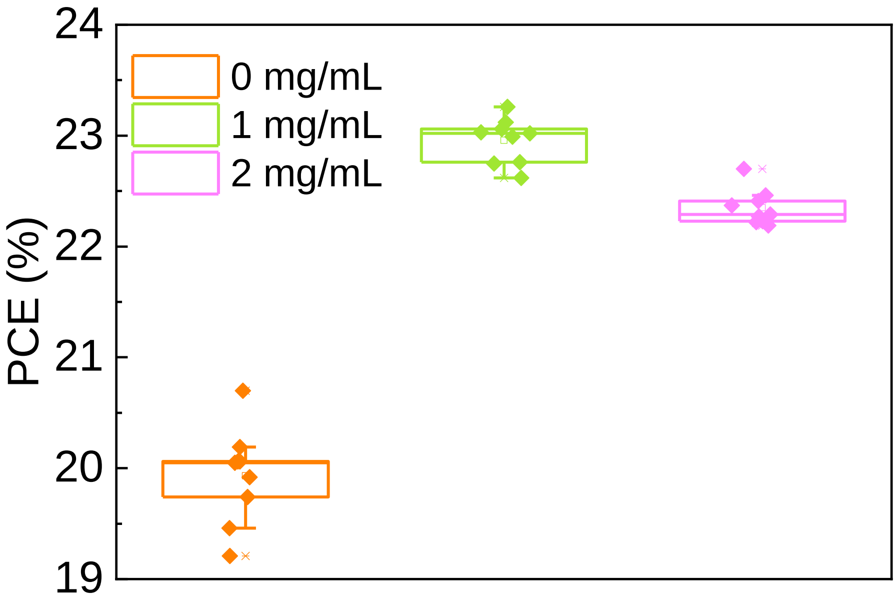

Supplementary Figure 19. Efficiency statistics of devices with *o*-CB concentrations of 0 mg mL^{-1}, 1 mg mL^{-1}, and 2 mg mL^{-1}.

The devices treated with 1 mg mL^{-1} of *o*-CB showed the best performance.

Supplementary Figure 20

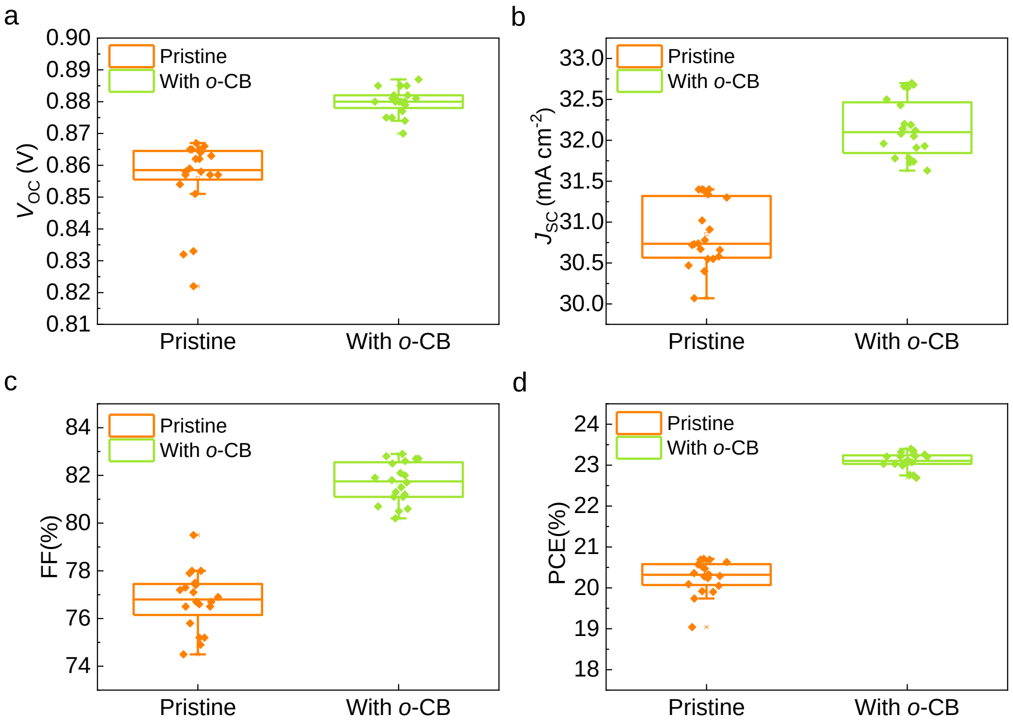

Supplementary Figure 20. Performance parameters statistics of devices with/without *o*-CB, **a)** V_{OC}, **b)** J_{SC}, **c)** FF, and **d)** PCE.

Supplementary Figure 21

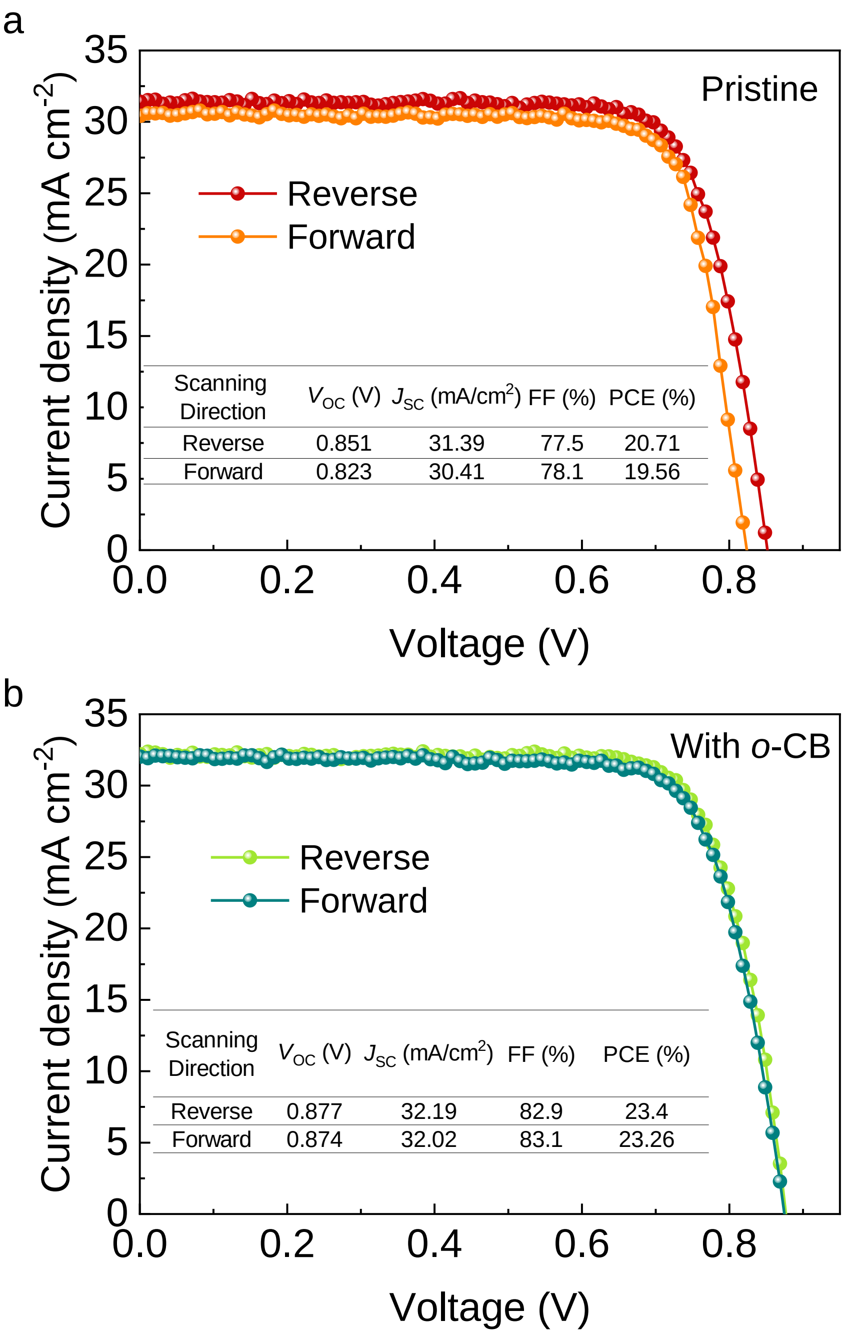

Supplementary Figure 21. *J*-*V* curves of forward and reverse scanning directions for **a)** Pristine and **b)** With *o*-CB devices.

After the *o*-CB treatment, the forward and reverse sweep hysteresis effect of the device was effectively suppressed.

Supplementary Figure 22

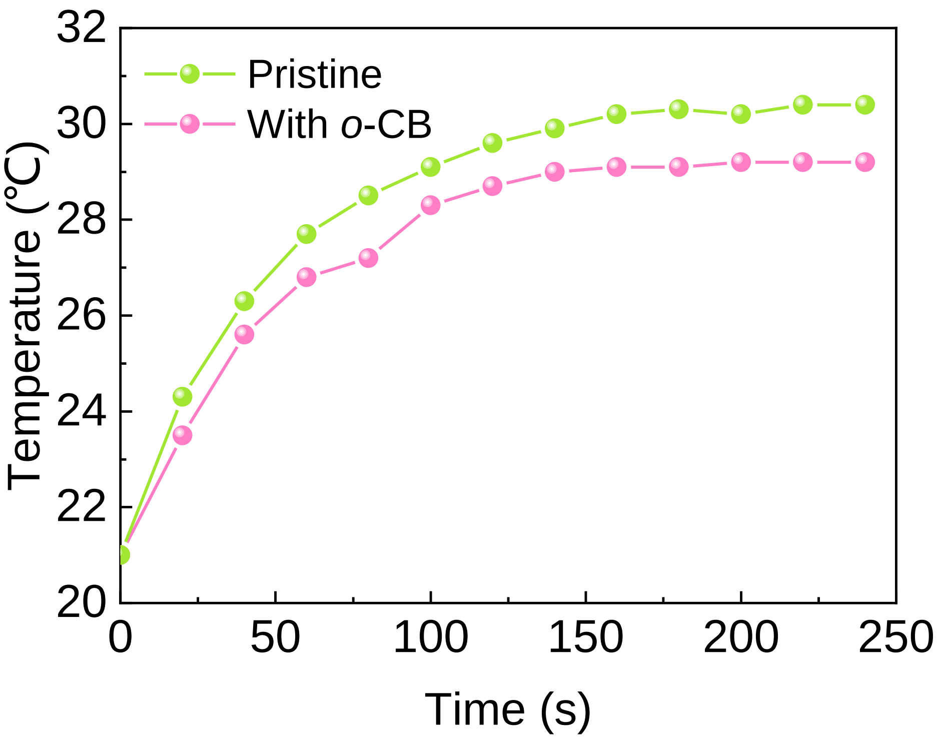

Supplementary Figure 22. Continuous recording of the temperature change of each layer of the device under illumination (AM 1.5G, 100 mW cm^{-2}).

To demonstrate that the improved crystallization of perovskite does not invalidate the increase of thermal conductivity due to the introduction of *o*-CB that leads to improved thermal stability of the device, we directly dropped the precursor solution with/without *o*-CB onto the substrate. We then placed them on a 100 °C hot plate for 15 minutes to allow the excess solvent to completely evaporate. After annealing, we connected the thin film with a T-type thermocouple and placed them under 100 mW cm^{-2} of AM 1.5G light for temperature tracking. The results are shown in the figure. For both poor crystalline films, the pristine perovskite film exhibited a continuously higher temperature than the film treated by *o*-CB upon heating.

Supplementary Figure 23

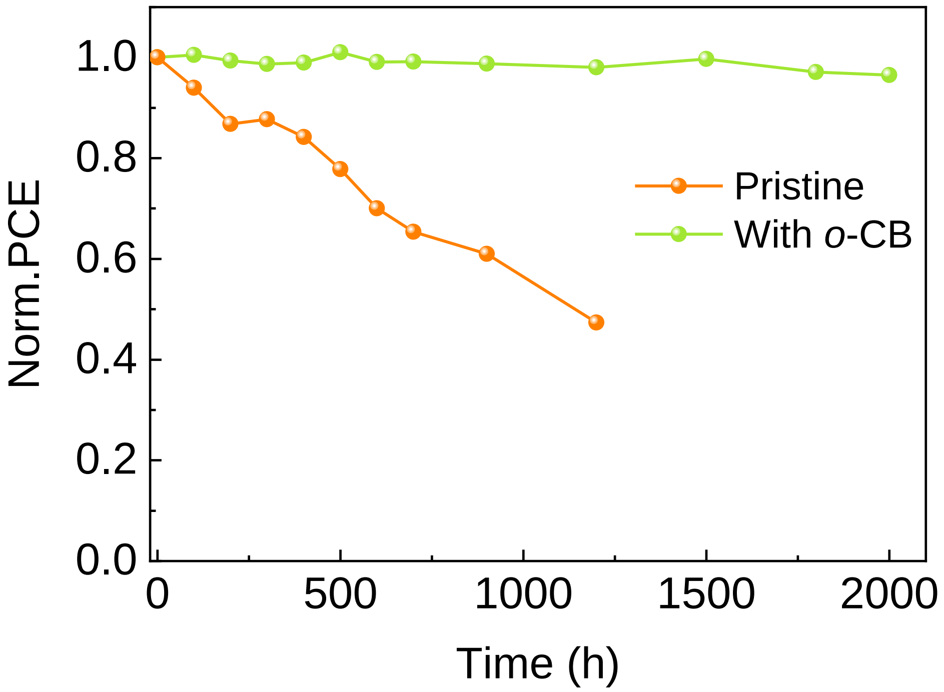

Supplementary Figure 23. Long-term stability of unencapsulated devices with/ without *o*-CB treatment in N_2 environment (~25 °C, 15% RH).

The efficiency of the device after *o*-CB treatment can still maintain 96 % of the initial value after 2000 h. The efficiency of the pristine device dropped to 46 % of its original efficiency at 1200 h.

Supplementary Figure 24

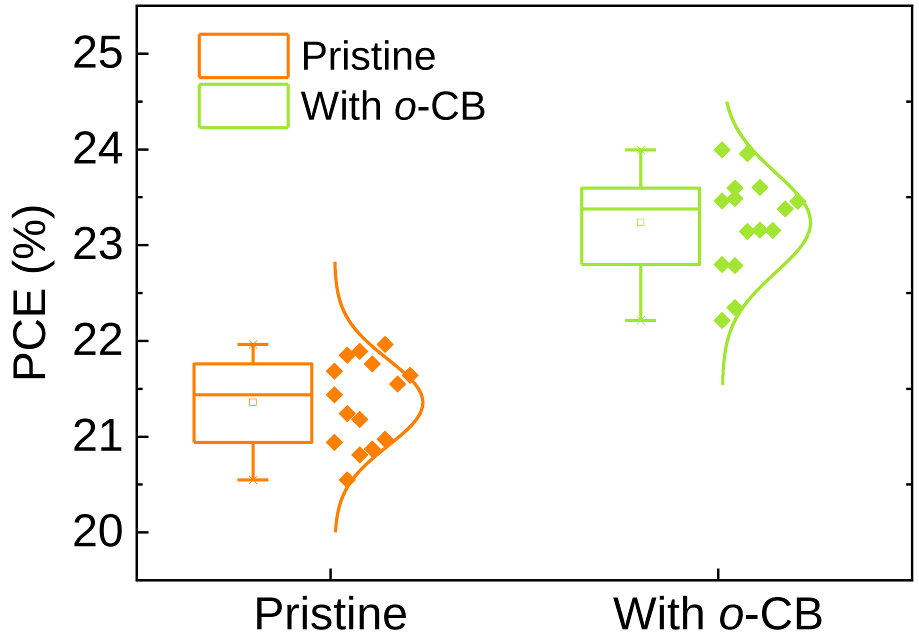

Supplementary Figure 24. Performance parameters statistics of devices with/ without *o*-CB.

Significantly improved efficiency of pure lead devices after *o*-CB treatment.

Supplementary Figure 25

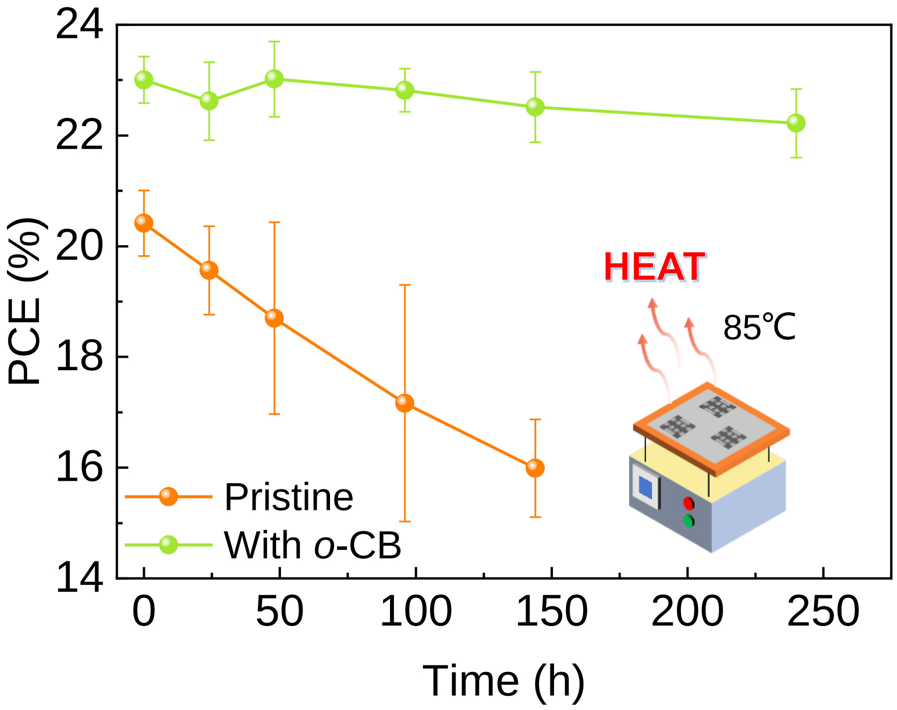

Supplementary Figure 25. Thermal stability of devices with pure lead perovskite systems at 85 °C. The error bars in the figure represent the standard deviation of 7 devices.

The efficiency of the *o*-CB-treated devices remained at 95 % of the initial values after 240 h. In comparison, the efficiency of the pristine devices decreased to 65 % of the original efficiency after 144 h.

Supplementary Figure 26

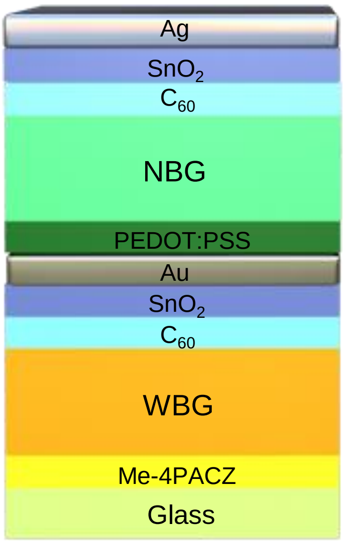

Supplementary Figure 26. The device structure of the all-perovskite tandem solar cells.

Following ITO/([4-(3,6-Dimethyl-9H-carbazol-9-yl)butyl]phosphonic Acid) Me-4PACz/$FA_{0.8}Cs_{0.2}Pb(I_{0.7}Br_{0.3})_3$ wide-bandgap perovskite absorber/C_{60}/ALD SnO_2/Au/PEDOT:PSS/$Cs_{0.1}MA_{0.2}FA_{0.7}Pb_{0.5}Sn_{0.5}I_3$ low-bandgap perovskite absorber/C_{60}/ALD SnO_2/Ag.

Supplementary Figure 27

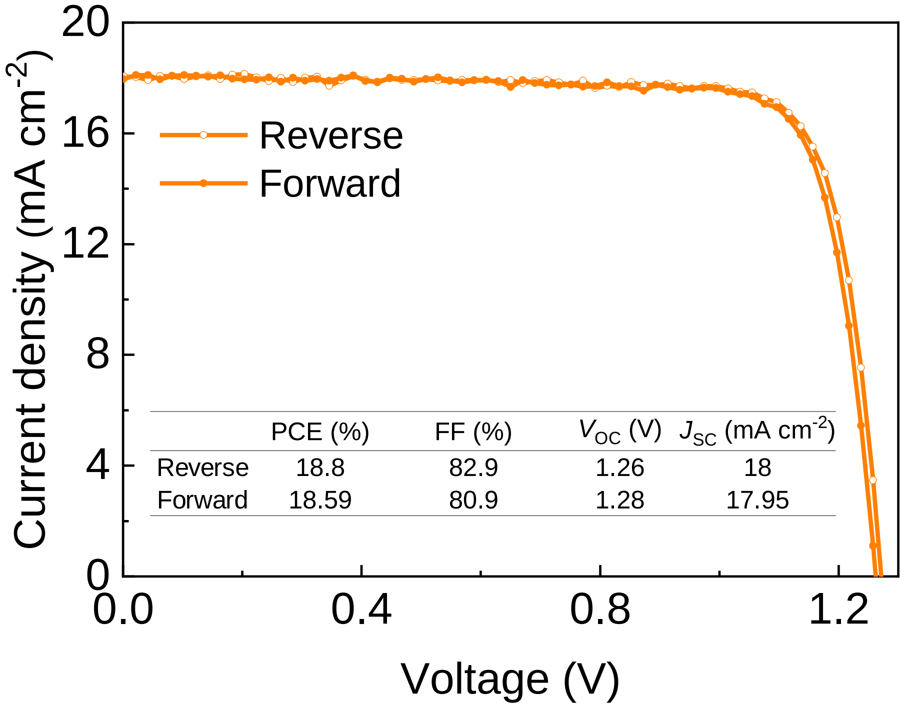

Supplementary Figure 27. *J*-*V* curves of the champion WBG PSC.

Supplementary Figure 28

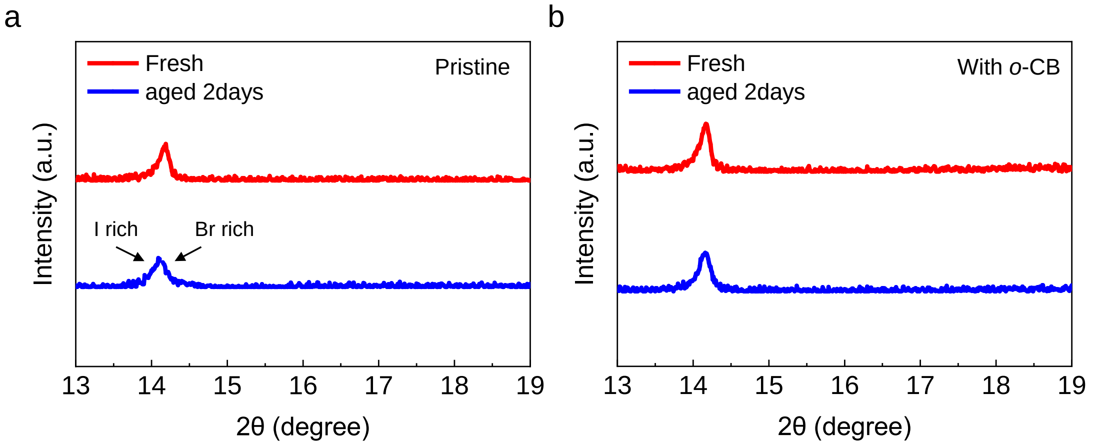

Supplementary Figure 28. XRD patterns of the perovskite films **a**) without and **b**) with *o*-CB treatment before and after 100 mW cm^{-2} white light illumination for 2 days.

It can be seen that the introduction of *o*-CB into the wide-bandgap perovskite films can suppress I-Br separation.

Supplementary Tables

Supplementary Table S1. Thermal conductivities (W m^{-1} K^{-1}) of mixed Sn-Pb perovskites w/ and w/o *o*-CB treatment.

	Pristine	With *o*-CB
25 ℃	2.476	2.697
55 ℃	2.441	2.592
85 ℃	2.325	2.486

Supplementary Table S2. The thermal resistance (R) and thermal transmissivity (K) of devices w/ and w/o *o*-CB.

Pristine	R (°C/W)	K (W/mk)	With *o*-CB	R (°C/W)	K (W/mk)
25°C	2.1782	1.4430	25°C	2.1167	1.4780
55°C	2.2031	1.4200	55°C	2.1379	1.4350
85°C	2.2225	1.3550	85°C	2.1641	1.3620

Supplementary Table S3. FWHM of (110) and (220) diffraction planes for perovskite films w/ and w/o *o*-CB treatment.

Perovskite film	FWHM (110)	FWHM (220)
Pristine	0.08651	0.07103
With *o*-CB	0.08596	0.06991

Supplementary Table S4. Fermi energy level of perovskite films w/ and w/o *o*-CB treatment obtained from VASP calculation.

Perovskite film	E_f (eV)
Pristine	-2.5331
With *o*-CB	-2.3379

Supplementary Table S5. TRPL spectral parameters of perovskite films w/ and w/o *o*-CB treatment.

Sample	A1/%	τ_1/ns	A2/%	τ_2/ns	τ_{ave}/ns
Pristine	87	49	13	544.6	358
With *o*-CB	75	99	25	2222	630

Supplementary Table S6. Photovoltaic parameters of the optimal devices w/ and w/o *o*-CB treatment.

Sample	V_{OC} (V)	J_{SC} (mA cm^{-2})	FF (%)	PCE (%)
Pristine	0.851	31.39	77.5	20.71
	0.862	31.34	75.2	20.32
	0.858	30.91	78	20.69
	0.822	31.4	79.5	20.52
	0.833	31.02	77.1	19.92
	0.863	30.66	76.7	20.29
	0.866	30.55	75.2	19.9
	0.857	31.3	76.9	20.63

Sample	V_{OC} (V)	J_{SC} (mA cm^{-2})	FF (%)	PCE (%)
With *o*-CB	0.877	32.19	82.9	23.4
	0.881	32.2	81.3	23.06
	0.885	31.0	83.7	22.99
	0.882	31.9	82.0	23.12
	0.874	31.7	82.0	22.75
	0.880	31.9	81.9	23.03
	0.875	31.8	82.8	23.02
	0.881	31.9	82.7	23.26

Supplementary References

1. Kresse, G. & Hafner, J. Ab initio molecular-dynamics simulation of the liquid-metal-amorphous-semiconductor transition in germanium. *Phys. Rev. B* **49**, 14251-14269 (1994).
2. Kresse, G. & Furthmüller, J. Efficient iterative schemes for ab initio total-energy calculations using a plane-wave basis set. *Phys. Rev. B* **54**, 11169-11186 (1996).
3. Kresse, G. & Furthmüller, J. Efficiency of ab-initio total energy calculations for metals and semiconductors using a plane-wave basis set. *Comput. Mater. Sci.* **6**, 15-50 (1996).
4. Perdew, J.P. et al. Generalized Gradient Approximation Made Simple. *Phys. Rev. Lett.* **77**, 3865-3868 (1996).
5. Blöchl, P.E. Projector augmented-wave method. *Phys. Rev. B* **50**, 17953-17979 (1994).
6. Monkhorst, H.J. & Pack, J.D. Special points for Brillouin-zone integrations. *Phys. Rev. B* **13**, 5188-5192 (1976).
7. Grimme, S. Semiempirical GGA-type density functional constructed with a long-range dispersion correction. *J Comput Chem* **27**, 1787-1799 (2006).
8. Tang, W. et al. A grid-based Bader analysis algorithm without lattice bias. *J. Phys. Condens. Matter* **21** (2009).
