# Supplementary material for: Sustainable thermal regulation improves stability and efficiency in all-perovskite tandem solar cells
Source: Nat Commun. 2024 May 16;15:4136. doi: 10.1038/s41467-024-48552-2 (PMC11099067; doi:10.1038/s41467-024-48552-2)
Supplement: Supplementary file 3 — Reporting Summary [file 41467_2024_48552_MOESM3_ESM.pdf]

nature portfolio

Corresponding author(s): Zhongmin Zhou

# Solar Cells Reporting Summary

Nature Portfolio wishes to improve the reproducibility of the work that we publish. This form is intended for publication with all accepted papers reporting the characterization of photovoltaic devices and provides structure for consistency and transparency in reporting. Some list items might not apply to an individual manuscript, but all fields must be completed for clarity.

For further information on Nature Research policies, including our data availability policy, see Authors & Referees.

## Experimental design

**Please check the following details are reported in the manuscript, and provide a brief description or explanation where applicable.**

1. Dimensions

Area of the tested solar cells
- [x] Yes — 0.09 cm2
- [ ] No — *Explain why this information is not reported/not relevant.*

Method used to determine the device area
- [x] Yes — Defined by the shadow masks
- [ ] No — *Explain why this information is not reported/not relevant.*

2. Current-voltage characterization

Current density-voltage (J-V) plots in both forward and backward direction
- [x] Yes — Supplementary Figure 21
- [ ] No

Voltage scan conditions
- [x] Yes — Device PV performance characterization section
- [ ] No — *Explain why this information is not reported/not relevant.*

Test environment
- [x] Yes — Device PV performance characterization section
- [ ] No — *Explain why this information is not reported/not relevant.*

Protocol for preconditioning of the device before its characterization
- [ ] Yes — *Provide a description of the protocol.*
- [x] No — No preconditioning is required for our cell characterization

Stability of the J-V characteristic
- [x] Yes — Steady-state efficiencies were obtained by tracking the maximum power point. Figure 4f, Figure 4i
- [ ] No — *Explain why this information is not reported/not relevant.*

3. Hysteresis or any other unusual behaviour

Description of the unusual behaviour observed during the characterization
- [x] Yes — Hysteresis was found in Control. Others show the negligible hysteresis
- [ ] No — *Explain why this information is not reported/not relevant.*

Related experimental data
- [x] Yes — Figure 4g, Table 1, Supplementary Figure 21, Supplementary Figure 27
- [ ] No — *Explain why this information is not reported/not relevant.*

4. Efficiency

External quantum efficiency (EQE) or incident photons to current efficiency (IPCE)
- [x] Yes — Figure 4c and Figure 4h
- [ ] No — *Explain why this information is not reported/not relevant.*

A comparison between the integrated response under the standard reference spectrum and the response measure under the simulator
- [x] Yes — Figure 4b and 4c, Figure 4h and supplementary Figure 27
- [ ] No — *Explain why this information is not reported/not relevant.*

| | | |
|---|---|---|
| For tandem solar cells, the bias illumination and bias voltage used for each subcell | ☒ Yes<br>☐ No | For tandem solar cells, EQE measurements were performed in ambient air and bias illumination from bright LEDs, with emission peaks of 850 and 450 nm used for measurement of the front and back subcells, respectively. No bias voltage was applied during EQE measurement of tandem solar cells.<br>*Explain why this information is not reported/not relevant.* |

## 5. Calibration

| | | |
|---|---|---|
| Light source and reference cell or sensor used for the characterization | ☒ Yes<br>☐ No | Device PV performance characterization<br>*Explain why this information is not reported/not relevant.* |
| Confirmation that the reference cell was calibrated and certified | ☒ Yes<br>☐ No | Device PV performance characterization<br>*Explain why this information is not reported/not relevant.* |
| Calculation of spectral mismatch between the reference cell and the devices under test | ☒ Yes<br>☐ No | A standard silicon wafer cell was used as the reference for the EQE measurement<br>*Explain why this information is not reported/not relevant.* |

## 6. Mask/aperture

| | | |
|---|---|---|
| Size of the mask/aperture used during testing | ☒ Yes<br>☐ No | 0.09 cm2<br>*Explain why this information is not reported/not relevant.* |
| Variation of the measured short-circuit current density with the mask/aperture area | ☐ Yes<br>☒ No | *Report the difference in the short-circuit current density values measured with the mask and aperture area.*<br>The short-circuit current density does not vary with the mask/aperture area |

## 7. Performance certification

| | | |
|---|---|---|
| Identity of the independent certification laboratory that confirmed the photovoltaic performance | ☐ Yes<br>☒ No | *Identify the independent certification laboratory.*<br>We focused on the sustainable thermal regulation, and we did not aim to claim a world record efficiency |
| A copy of any certificate(s) | ☐ Yes<br>☒ No | *Certificate copies should be provided in the Supplementary information. Please state the supplementary item number.*<br>We focused on the sustainable thermal regulation, and we did not aim to claim a world record efficiency |

## 8. Statistics

| | | |
|---|---|---|
| Number of solar cells tested | ☒ Yes<br>☐ No | For Sn/Pb, 20 devices were fabricated and measured (Supplementary Figure 20). For Pb, 15 devices were fabricated and measured (Supplementary Figure 24)<br>*Explain why this information is not reported/not relevant.* |
| Statistical analysis of the device performance | ☒ Yes<br>☐ No | Table 1, Supplementary Figure 20, Supplementary Figure 24, Supplementary Table S6<br>*Explain why this information is not reported/not relevant.* |

## 9. Long-term stability analysis

| | | |
|---|---|---|
| Type of analysis, bias conditions and environmental conditions | ☒ Yes<br>☐ No | The stability was tested in the ambient in air and at room temperature by tracking the steady state efficiency of the device under continuous 100 mW cm-2 AM 1.5G solar irradition (Figure 4f and 4i)<br>*Explain why this information is not reported/not relevant.* |
